# Recent advances in understanding DNA methylation of prostate cancer

**DOI:** 10.3389/fonc.2023.1182727

**Published:** 2023-05-10

**Authors:** Hyun Jin Shin, Junjie T. Hua, Haolong Li

**Affiliations:** ^1^ Helen Diller Family Comprehensive Cancer Center, University of California, San Francisco, San Francisco, CA, United States; ^2^ Department of Radiation Oncology, University of California, San Francisco, San Francisco, CA, United States

**Keywords:** epigenetic regulation, DNA methylation, 5mC, CIMP, mCRPC

## Abstract

Epigenetic modifications, such as DNA methylation, is widely studied in cancer. DNA methylation patterns have been shown to distinguish between benign and malignant tumors in various cancers, including prostate cancer. It may also contribute to oncogenesis, as it is frequently associated with downregulation of tumor suppressor genes. Aberrant patterns of DNA methylation, in particular the CpG island hypermethylator phenotype (CIMP), have shown associative evidence with distinct clinical features and outcomes, such as aggressive subtypes, higher Gleason score, prostate-specific antigen (PSA), and overall tumor stage, overall worse prognosis, as well as reduced survival. In prostate cancer, hypermethylation of specific genes is significantly different between tumor and normal tissues. Methylation patterns could distinguish between aggressive subtypes of prostate cancer, including neuroendocrine prostate cancer (NEPC) and castration resistant prostate adenocarcinoma. Further, DNA methylation is detectable in cell-free DNA (cfDNA) and is reflective of clinical outcome, making it a potential biomarker for prostate cancer. This review summarizes recent advances in understanding DNA methylation alterations in cancers with the focus on prostate cancer. We discuss the advanced methodology used for evaluating DNA methylation changes and the molecular regulators behind these changes. We also explore the clinical potential of DNA methylation as prostate cancer biomarkers and its potential for developing targeted treatment of CIMP subtype of prostate cancer.

## 1 Introduction

Prostate cancer is the most common cancer in men, making up 29% of new cases, and one of the top cancer-related causes of death in men in the United States (1). In prostate cancer, androgen receptor (AR) is a key oncogenic driver, is often found amplified in the gene body and enhancer upstream of AR and is associated with aggressive progression of disease. While androgen-deprivation therapy (ADT) is the first line treatment for patients with locally advanced or metastatic prostate cancer, the disease often progresses to the castration resistant prostate cancer (CRPC) stage. Newly developed AR targeted therapies, such as enzalutamide and abiraterone, have shown promise as effective treatments for advanced prostate cancer. Yet, the resistant tumor invariably occurs, leading the disease to its terminal stage. To understand disease progression and treatment resistance, majority of studies have focused on genetic alterations of the key drivers such as *AR* gene, *AR* co-factors (e.g. *NCOA2*, *EP300*, and *FOXA1*), *ETS* gene fusions (e.g. *TMPRSS2-ERG* fusion), *SPOP* mutations, mutations affecting gene expression, and chromatin regulation (e.g. *KDM6A/UTX*, *MLL2*, *MLL3*, *CHD1*, and *EZH2*) *(* 2, 3). Some of these genetic changes can be used as prognostic markers, such as mutations in *BRCA1*, *BRCA2*, *HOXB13*, *ATM*, and *CHEK2*, dysregulation of *PTEN*, *TMPRSS2-ERG* fusion, and high overall number of somatic copy number aberrations, which are associated with poor prognosis (4). In addition to *AR* signaling driven tumors, subtypes such as neuroendocrine prostate cancer (NEPC) are *AR*-independent, thus making ADT ineffective (5). NEPC is an aggressive histologic subtype of prostate cancer associated with poor prognosis. NEPC tumors share some common genetic aberrations as prostate adenocarcinoma, such as *TMPRSS2-ERG* fusion and loss of *RB1* and *TP53*, but often do not express *AR* and downstream *AR*-regulated targets such as PSA and prostate-specific membrane antigen (PSMA) (6). Beyond genomic and transcriptomic subtypes of prostate cancer, epigenetic alterations are also found to play a critical role in prostate cancer progression and treatment resistance.

DNA methylation is an epigenetic process that involves the addition of methyl groups to DNA. The most predominant type of DNA methylation, termed 5-methylcytosine (5mC), typically happens on cytosines of CpG dinucleotide sequences and are usually associated with DNA inactivation. DNA methylation is modulated by DNA methyltransferase (DNMT) and ten eleven translocation (TET) enzymes, which are writers and erasers of 5mC, respectively. More specifically, DNMT1 maintains DNA methylation and prefers hemimethylated DNA, and DNMT3A and DNMT3B are responsible for *de novo* DNA methylation. Meanwhile, the TET family enzymes, TET1, TET2, and TET3, convert 5mC back to unmethylated cytosine in a series of steps with functionally important intermediates. 5mC is first converted to 5-hydroxymethylcytosine (5hmC), then 5-formylcytosine, followed by 5-carboxylcytosine, and finally back to cytosine. A portion of total methylation modifications consists of 5hmC. Unlike 5mC, 5hmC is enriched at transcriptionally active regions and associated with expression of many genes. Further, IDH1 and IDH2, which are not directly involved in methylation, modulates methylation by affecting TET2 function. IDH1 and IDH2 produces α-ketoglutarate, an obligatory substrate for TET. Most CpG dinucleotides in the genome are highly methylated, with the exception of CpG islands, CpG shores ( ± 2 kbp around islands), and CpG shelves ( ± 2kbp around shores), which show variable methylation level (7). CpG islands are regions of DNA with high concentration of CpG dinucleotides and are typically associated with cis-regulatory regions such as promoters. DNA methylation at promoters leads to gene repression by blocking transcription factors from binding and/or by recruiting methyl-CpG binding domain proteins that subsequently recruit and synergize with chromatin remodelers and histone deacetylases to establish a silenced chromatin state for long-term transcriptional repression (8–10). Similarly, DNA methylation at enhancers is reported to repress enhancer activity (11). While DNA methylation is essential for mammalian development and aging, aberrant methylation patterns are significant contributors to oncogenesis (12). Specifically, global DNA hypomethylation and hypermethylation of specific CpG islands is frequently observed in cancer, leading to expression of normally silenced repetitive elements and repression of tumor suppressors and DNA repair genes, respectively (13–15). Interestingly, these CpG hypermethylation profiles are found to be highly tumor-type-specific and may serve as potential biomarkers (15). By clustering cancer samples based on methylation levels at specific loci, a subtype of tumors characterized by hypermethylation of CpG island methylation has been identified and termed the CpG island methylator phenotype (CIMP).

The presence of a CIMP subtype is widely accepted in several cancer types, such as colorectal and breast cancer. However, whether CIMP is a pan-cancer phenomenon is still unclear, as are the exact molecular mechanisms driving CIMP (16). Most early findings on CIMP have been solely based on selective loci that lack consistencies between studies and cancer types, which hindered pan-cancer interpretation. Recent improvement of sequencing technologies and development of novel sequencing approaches, particularly whole-genome bisulfite sequencing, plays a significant role in providing a pan-cancer CIMP definition (17). Furthermore, integrative analyses across different sequencing approaches have accelerated our understanding of potential molecular mechanisms behind CIMP. Clinically, CIMP subtype is often associated with differential tumor prognosis, aggressiveness, and survival across different cancer types, which highlights the potential for the development of methylation-based prognostic biomarkers. Furthermore, demethylating agents are showing promise as novel cancer treatments (16, 18, 19). However, whether CIMP-associated hypermethylation is causal in tumorigenesis and cancer progression has remained largely elusive until recently (20). Further, other epigenetic changes are believed to be signs of disease progression in prostate cancer. For instance, changes like chromatin accessibility, SWI/SNF, histone marks, and DNA methylation, are distinguishing features of NEPC (5). This review will highlight recent biological and clinical findings of CIMP and other changes in methylation patterns in prostate cancer and discuss its clinical potentials.

## 2 DNA methylation and cancer

Aberrant DNA methylation patterns, specifically CIMP, is well established in multiple cancers, including colorectal cancer, gastric cancer, glioma, breast cancer, and leukemia. CIMP has first been identified in colorectal cancer through the detection of colorectal cancer-specific methylation in selective CpG island regions, including *p16* and *THBS1 (* 18). This concept has subsequently been validated by various studies which examined additional regions (21–25). CIMP is now a well-established molecular subtype of colorectal cancer that is associated with specific genetic and clinicopathological features and tumorigenic pathways. Notably, CIMP colorectal cancer is associated with *BRAF V600E* and *KRAS* mutations, *CDX2* loss, as well as low chromosomal aberrations and high microsatellite instability (MSI), which is reported to be driven by methylation of *hMLH1* gene (18, 21, 23–26). Colorectal cancer can be further categorized into three molecularly distinct subclasses based on CIMP status: CIMP-high (CIMP-H) tumors associated with MSI and *BRAF* mutations, CIMP-low tumors associated with *KRAS* mutations, and CIMP-negative tumors associated with high *p53* mutations (20, 23). However, beyond these associations, the causal relationship between CIMP and these key driver mutations in colorectal cancer are largely unclear. Recent studies have found that aberrant DNA methylation occurs at early stages of colorectal cancer development and may sensitize colorectal cells to *BRAF V600E*-driven tumorigenic transformations into colorectal cancer (25, 26).

CIMP has been identified in other types of cancer but lacks a clear definition. In gastric cancer, CIMP is identified based on hypermethylation of specific genes, most commonly *MINT1*, *MINT2*, *MINT12*, *MINT25*, *MINT31*, *hMLH1*, and *p16 (* 27–34). While many studies’ CIMP markers include these genes, others do not; and more recent studies use whole-genome sequencing to identify CIMP. Meanwhile, CIMP is identified in breast cancer by hypermethylation of a few genes, including tumor suppressor genes *BRCA1*, *p16*, *APC (* 35, 36). Moreover, in blood cancer, CIMP has been initially identified in acute myeloid leukemia and acute lymphoblastic leukemia based on hypermethylation of specific genes such as *CDH1*, *CDH13*, and *sFRP1 (* 37, 38). However, these sites, as well as few additional CpG sites, have only been validated in some but not all studies, suggesting extensive heterogeneity between studies (39–41). Overall, there has also been a lack of consensus on the definition of CIMP in leukemia in terms of which specific CpG sites were used as biomarkers to identify CIMP. More recently, several studies have applied genome-wide DNA methylation arrays along with a panel of 1,293 CpG sites to classify CIMP in leukemia to standardize the field (42, 43). Using this approach, CIMP has been defined by comparing methylation levels across a wider range of CpG sites rather than a methylation of specific genes, providing a clearer definition of CIMP. Overall, there is a lack of consensus on the definition of CIMP in various cancers in terms of specific sites used as CIMP markers, but new methods of detecting methylation allow identification of CIMP based on methylation of a greater number of CpG islands.

Despite the lack of a consistent definition of CIMP across cancer types in early studies, there is a consensus between multiple studies within each cancer type that CIMP is associated with distinct molecular features (**Table 1**). In gastric cancer, CIMP is associated with MSI, Epstein-Barr virus (EBV)-associated gastric cancer, and *H. pylori* infection (27, 28, 32–34, 44–47). Unlike colorectal cancer CIMP, gastric CIMP is not associated with *p53* and *KRAS* mutations (28, 32, 48, 49). Methylation patterns in breast cancer show association with molecular subtypes of breast cancer. Most notably, CIMP is associated with the presence of estrogen receptor (ER) and progesterone receptor (PR) status (36, 50, 51). CIMP is also associated with invasive lobular breast cancer, which displays higher frequency of hypermethylation than invasive ductal carcinoma (52, 53). Also, CIMP shows association with copy number alterations, which could help further classify breast cancer subtypes (53, 54). CIMP in leukemia is associated with distinct molecular features, including *TET2*, *IDH1* and *IDH2* mutations in acute myeloid leukemia (43, 55–58). Furthermore, in acute myeloid leukemia, CIMP can be further divided into two categories, I-CIMP associated with *IDH1/2* mutations and A-CIMP enriched in *CEBPA* and *WT1* mutations (59). These molecular features suggest an association between hypermethylation and leukemogenesis, but a causal relationship has yet to be established. Moreover, in glioma, hypermethylation of promoter associated CpG islands of genes such as *TMS1/ASC* is commonly reported (60–62). CIMP has then been identified in grade IV gliomas, or glioblastomas, and in lower grade gliomas and found to be associated with specific molecular and clinical features (63–65). Significantly, CIMP is associated with methylation of O^6^-methylguanine DNA methyltransferase (*MGMT*) gene and *IDH1* mutations (63, 65–67). Also, a study has found IDH1 can induce DNA hypermethylation that mimics CIMP subtypes in lower grade gliomas, suggesting a causal relationship (65). In addition, CIMP status is also associated with gene copy variations (68, 69).

**Table 1:** Hypermethylated genes and associated molecular features of CIMP in various cancer types.

Cancer Type	Frequently hypermethylated genes	Associated molecular features
Colorectal cancer	• *P16* • *THBS1*	• *BRAF V600E* and *KRAS* mutations • *CDX1* loss • Low chromosomal aberrations • Microsatellite instability (MSI)
Gastric cancer	• *MINT1* • *MINT2* • *MINT12* • *MINT25* • *MINT31* • *hMLH1* • *p16*	• MSI • Epstein-Barr virus (EBV)-associated gastric cancer • *H. pylori* infection
Breast cancer	• *BRCA1* • *P16* • *APC*	• Presence of estrogen receptor and progesterone receptor status • Invasive lobular breast cancer • Copy number alterations
Acute myeloid leukemia and lymphoblastic leukemia	• *CDH1* • *CDH13* • *sFRP1*	• *TET2*, *IDH1*, and *IDH2* mutations • I-CIMP associated with *IDH1/2* mutations • A-CIMP enriched in *CEBPA* and *WT1* mutations
Glioma	• *TMS1/ASC*	• Methylation of *MGMT* • *IDH1* mutations • *Gene copy variations*
Prostate cancer	• *RARβ* • *GSTP1* • *CDH13* • *RASSF1A* • *APC* • *p16* • *DAPK* • *FHIT* • *MGMT* • *CDH1*	• Significantly higher methylation levels at recurrent hypomethylated regions in mCRPC • RNA expression of oncogenic driver genes such as *AR*, *MYC*, and ERG in mCRPC • Less likely to have *ETS* fusions or *TP53* biallelic inactivation in mCRPC • Mutations in *TET2*, *IDH1*, *BRAF*, and *DNMT3B* in mCRPC • Downregulation of tumor suppressor genes

## 3 DNA methylation in prostate cancer

### 3.1 Methods for DNA methylation detection

Methylation-specific PCR (MS PCR) was most commonly used in early studies to analyze DNA methylation patterns in CpG island and determine CIMP status. For MS PCR, DNA is first purified and treated with sodium bisulfite, which converts cytosine cytosine to uracil but not 5-methylcytosine. Then, PCR is performed with two primer pairs for detectable methylated and unmethylated DNA. A different method to analyze DNA methylation patterns is using methyl-CpG binding domain (MBD). MBD preferentially binds methylated DNA and can be used to enrich methylated genomic DNA fragments and create libraries (70). This library can be analyzed using real-time PCR, tiling microarrays, and next-generation sequencing. DNA methylation alterations in prostate cancer samples can also be analyzed by sequencing sodium-bisulfite-converted genomic DNA (e.g. Illumina HumanMethylation27, MethylPlex-next-generation sequencing, MethylationEPIC Bead-Chip), which allows more quantitative accuracy and detection sensitivity, high efficiency, and a wide spectrum for analysis (71). Similar to MS PCR, for this method, DNA is treated with sodium bisulfite, then subsequent PCR and specific methylation primers are used to sequence and identify the methylated genomic regions (71). Another method to analyze DNA methylation patterns is using methylated DNA immunoprecipitation sequencing (MeDIP). In this method, genomic DNA is sonicated and immunoprecipitated using antibodies specific to 5mC, and resulting fragments are amplified, prepped, and sequenced. Similarly, hydroxymethylated DNA immunoprecipitation sequencing (hMeDIP-seq) can be used to analyze patterns of hydroxymethylation, using the same methods as MeDIP-seq, but using antibodies specific to 5hmC instead.

DNA methylation analysis methods each have their strengths and limitations. For example, array-based methods are useful for profiling DNA methylation changes across large regions of the genome, but they have limited coverage of CpG sites (72). Reduced representation bisulfite sequencing (RRBS) can provide high coverage of CpG sites, but its ability to read the entire genome is limited. Whole-genome bisulfite sequencing (WGBS) offers the highest resolution and can be performed on single nuclei, but the method would not distinguish 5mc and 5hmc (73). A combined 5mc and 5hmc detection method such as WGBS and oxidative WGBS (oxWGBS) could provide a more comprehensive view of DNA methylation changes, but this has not yet been extensively studied in the context of prostate cancer.

### 3.2 Patterns of CIMP in prostate cancer

Patterns in DNA methylation in prostate cancer are distinct from other cancer types mentioned above but share some common features. CIMP in prostate cancer is often determined by checking methylation status of several loci using MS PCR, then later confirmed using bilsulfite DNA sequencing (74). With newer methods of detecting DNA methylation at a more global scale, CIMP can be determined by cancer-specific differentially methylated regions rather than through checking DNA methylation of specific genes. As in leukemia, CIMP in prostate cancer can be identified as groups with higher methylation levels when comparing these differentially methylated regions. Genes commonly hypermethylated in prostate cancer include tumor suppressor genes involved in DNA damage repair, cell adhesion, apoptosis, cell cycle control, signal transduction, and hormonal responses, such as *RARβ*, *GSTP1*, *CDH13*, *RASSF1A*, *APC*, *p16*, *DAPK*, *FHIT*, *MGMT*, and *CDH1 (* 70, 74–78). Some hypermethylated genes in prostate cancer are also hypermethylated in other types of cancer, such as *p16* in colorectal cancer (18), gastric cancer (34), and leukemia; and *CDH13*, *APC*, and *CDH1* in leukemia (35–38). When compared to samples from benign prostatic hyperplasia (BPH) and nonmalignant tissues, samples from prostate cancer showed higher levels of methylation (74, 76). Further, methylation of tumor suppressor genes *GSTP1*, *APC*, and *MGMT* is strongly associated with their downregulation, suggesting an important role for DNA methylation in driving carcinogenesis and disease progression (70, 78). Because increase in methylation may be an age-related event, Kang et al. have examined methylation status of *APC*, *COX2*, *DAPK*, *CDH1*, *GSTP1*, *MGMT*, *p14*, *p16*, *RASSF1A*, *RUNX3*, and *THBS1* from non-neoplastic prostate samples of mostly older men. They have found that there is very low or no promoter methylation in these samples, suggesting hypermethylation of specific loci in prostate cancer is likely not an age-related event, but rather a tumor-related one (78).

While initial studies on methylation in prostate cancer have been limited due to the focus on evaluating methylation levels through MS PCR of select loci in a small number of prostate cancer samples, newer methods of analyzing methylation levels have emerged and allowed to analyze methylation in prostate cancer in a broader spectrum of an increasing number of tumor samples. Using the MBD approach, Aryee et al. have generated a library of methylated genomic DNA fragments and hybridized the library to Affymetrix SNP 6.0 high-density oligonucleotide microarrays and found DNA methylation alterations are maintained across all metastases within the same individual, and that regions with high consistency of hypermethylation across metastases within individuals show enrichment for cancer-related genes (70). Variability in genome-wide methylation patterns in benign, low-grade, and high-grade prostate cancer have also been analyzed using MDB-isolated genome sequencing (MiGS). This has revealed variations in methylation patterns that can distinguish between benign, low-grade, and high-grade prostate cancer samples. Further, by integrating DNA methylation data with RNA-seq and survival data, they have shown hypermethylation regions are in gene promoters and at intergenic regions that are enriched for DNA-protein binding sites (79). In addition, they have shown that downregulation of genes where DNA methylation and expression are well correlated is associated with poor outcome (79).

Using the sodium-bisulfite sequencing method, it has been shown that methylation pattern alterations are more frequent in prostate cancer and in benign prostate tissues adjacent to tumor, compared to age-matched organ-donor prostates (80). In addition, overall promoter CpG island methylation is significantly increased in localized and metastatic cancer tissues, and differentially methylated regions are cancer-specific (81). Also, by profiling DNA methylation in plasma samples of patients with metastatic prostate cancer over 9 months, Silva et al. show that methylation patterns within an individual are consistent with clinical progression, including disease progression and therapeutic response (82). By integrating methylome analysis with whole genome sequencing (WGS) and transcriptome sequencing (mRNA-seq), Gerhauser et al. describe four molecular subgroups of prostate cancer of different aggressiveness. Subgroup 1 represents normal basal and luminal prostate epithelium. Subgroup 2 is associated with high immune cell content but low T-luminal cell content, high GS, and shorter time to biochemical recurrence. Subgroup 3 represents an intermediate-risk group, and Subgroup 4 is associated with a high fraction of normal-like luminal cells and a known gene signature associated with less-aggressive prostate cancer (83). Hypermethylator phenotype has also been identified in metastatic castration resistant prostate cancer (mCRPC) using whole-genome bisulfite sequencing paired with deep whole-genome and transcriptome sequencing (84). This subtype has significantly higher methylation levels at recurrent hypomethylated regions (HMRs) and overall fewer HMRs at CpG island, shores, shelves, and in CpG open seas (regions outside islands, shelves, and shores) (84). There is also increased hypermethylation at differentiation and cancer genes (70). More specifically, in mCRPC, methylation is associated with RNA expression of oncogenic driver genes such as *AR*, *MYC*, and *ERG (* 84). Moreover, key AR-associated genes, such as KLK3, NKX3-1, and FOLH1 are correlated with DNA methylation independent of DNA changes (84). They also have found that this subtype of tumors is less likely to have *ETS* fusions or *TP53* biallelic inactivation, is not significantly associated with anatomic site of biopsy, and contains mutually exclusive mutations in *TET2*, *IDH1*, *BRAF*, and *DNMT3B (* 84). In another study using enhanced reduced-representation bisulfite sequencing (eRRBS) on patient tumor samples, Beltran et al. show there is a strong epigenetic segregation between castration resistant neuroendocrine prostate cancer and castration resistant prostate adenocarcinoma. Notably, they have found hypermethylation and reduced expression of *SPDEF*, a tumor suppressor gene, in castration resistant neuroendocrine prostate cancer. This has been validated in the neuroendocrine prostate cancer cell line NCI-H660, as compared to prostate adenocarcinoma cell line LNCaP (85).

Furthermore, MeDIP sequencing of 51 tumor and 53 benign prostate samples has revealed there are more than 147,000 cancer-associated epigenetic alterations, there are significant global methylation pattern differences associated with *TMPRSS2-ERG* rearrangement status, and hypermethylation of miR-26a can be involved in *ERG* rearrangement-independent *EZH2* activation (86). Further, another study using the same technique on samples from plasma DNA of patients with localized and metastatic prostate cancer has found that there is global hypermethylation in metastatic samples and hypomethylation in the pericentromeric regions (87). It also has shown that there is hypermethylation of the promoter of *NR3C1*, a glucocorticoid receptor gene, that is associated with decreased immune signature (87).

Beyond the hypermethylator phenotype, there are other methylation changes that may hold significance in prostate cancer. For example, somatic mutations and putative regulatory regions are frequently located in regions that are differentially hypomethylated (84). Not only is methylation silencing of tumor suppressors a significant event in progression of cancer, cancer-associated hypomethylation in oncogenic genes leading to their overexpression in mCRPC is also important (84). Multiple expression associated HMRs (eHMR) have been identified near AR, including AR promoter, AR enhancer, and additional loci upstream and downstream of AR (84). Although AR promoter is hypomethylated in all tissues, other eHMR are only identified in mCRPC samples but not in benign or primary PCa samples (84). The number of these hypomethylated eHMR loci is positively associated with AR expression. Additionally, eHMR loci found in AR gene body is positively associated with AR expression, representing novel intergenic regulatory regions of AR that can potentially contribute to ADT-resistance (84). 5hmC levels have also been shown to be associated with various clinical features of prostate cancer using hMeDIP-seq. 5hmC marks activation of cancer drivers and downstream targets such as *AR*, *EZH2*, *CDK1*, *TBX3*, *HOXA13*, *FOXA1*, and *HOXB13 (* 88). There is also a progressive increase in 5hmC levels in genes in proliferative and oncogenic pathways during tumor progression, and 5hmC patterns can accurately track dedifferentiation and lineage plasticity to neuroendocrine and gastrointestinal lineages (88). Further, 5hmC patterns in cell-free DNA are able to be detected and used to accurately estimate ct-fraction and find specific gene activation of driver genes *TOP2A* and *EZH2* that are not altered at the DNA level (88). Overall, in addition to patterns in 5mC methylation, hypomethylation and 5hmC patterns show potential to be used as a prognostic biomarker that can differentiate various subtypes of prostate cancer that genetic changes alone cannot.

## 4 Molecular drivers of CIMP

The development of CIMP in cancer has been attributed to various genomic and environmental factors, which differs depending on the cancer type. Most notably, protein-coding mutations in *BRAF* and *IDH1* have been shown to establish CIMP (20, 65). In the case of CIMP-high colorectal cancer, spontaneous aging-like promoter hypermethylation makes organoids more sensitive to transformation by *BRAF V600E* mutation, which leads to CIMP (20). *BRAF V600E* mutation may lead to CIMP in a pathway that involves MAFG, which binds promoters of *MLH1* and other CIMP-related genes and recruits corepressor complex, leading to hypermethylation and gene silencing (89). In CIMP-low colorectal cancer, KRAS upregulates zinc-finger DNA-binding protein, ZNF304, which binds promoters and recruits a corepressor complex with DNMT1, leading to DNA hypermethylation (90). Contrastingly, in glioma and leukemia, *IDH1* mutations that result in 2-hydroxyglutarate production disrupts TET2 function and establishes CIMP and global DNA hypermethylation (58, 65). TET2 loss of function mutation itself is also associated with similar epigenetic defects as *IDH1* mutants, and *TET2* knockouts are also frequently associated with hypermethylation (58, 91–93). In addition, mutations in *DNMT3A* and *DNMT3B* and knock outs are also frequently associated with hypomethylation, while overexpression of *DNMT3B* is associated with hypermethylation in gastric and breast cancer cell lines (91, 94–100). As mentioned previously, mCRPC tumors of the hypermethylator subtype contain mutually exclusive mutations in *TET2*, *IDH1*, *BRAF*, and *DNMT3B*, suggesting mutations in these proteins may contribute to hypermethylation (84). Further, Kobayashi et al. have shown there is increased expression of *DNMT3A2*, *DNMT3B*, and *EZH2* in tumors, and transient *DNMT3B1* and *DNMT3B2* overexpression in primary prostate cells results in increased methylation of some CpG sites that show increased methylation in tumors (101). Furthermore, in AML, TET2, IDH1, and DNMT3B do not seem to affect each other in terms of methylation pattern and regulation of downstream genes, but IDH1 and DNMT3A do (58, 102). More specifically, co-occurrence of *DNMT3A* and *IDH1* mutations show epigenetic patterns different from those of either *IDH1* or *DNMT3A* mutation, upregulation of RAS signaling and unique sensitivity to MEK inhibition and appear to be associated with either worse clinical outcome or show no difference in EFS or OS (103–105). In addition, DNMT3A and TET2 also seem to affect one another, showing different methylation patterns and phenotypes (106, 107). However, it is not clear if this is the case in prostate cancer, especially considering Zhao et al. have found mutations in *TET2*, *IDH1*, and *BRAF* were mutually exclusive in mCRPC samples. While mutations in *TET2*, *IDH1*, *DNMT3A*, and *DNMT3B* may play a role in establishing distinct methylation patterns found in prostate cancer, further study is required to establish a causal relationship and see how the various proteins involved in methylation interact with and affect one another.

Aside from gene mutations, several other factors such as EBV infection, aging and hypoxia also contribute to methylation changes. In multiple studies, EBV infection of epithelial cells *in vitro* directly induces global hypermethylation of the host genome, around the transcription start site, and results in gene silencing (108). It is partly driven by EBV latency protein, latent membrane protein 2A (LMP2A), which upregulates expression of *DNMT1* and downregulates TET1 and TET2, as well as LMP1, which upregulates *DNMT1*, *DNMT3A*, and *DNMT3B (* 108). In addition, it has been previously demonstrated that aging is associated with increased CpG island hypermethylation in colon mucosa (18, 109). Methylation of CpG island on the ER gene becomes progressively more pronounced with age, even in the early stages of tumor formation (109). This methylation of CpG island is associated with transcription repression, and in some cases, less *ER* expression (109). Moreover, re-expression of ER gene showed growth inhibition of colon carcinoma cells (109). Together, these findings suggest reduced ER expression, which is associated with age-related hypermethylation of CpG island on the *ER* gene, may be an early event that predisposes to sporadic colorectal tumorigenesis. Furthermore, hypoxia has been shown to increase hypermethylation at gene promoters in murine breast tumors (110). Mechanistically, hypoxia inhibits oxygen-dependent catalytic activities of the TET family methylation erasers, leading to the accumulation of methylation (110, 111). Oxidative stress from inflammation can also induce CpG island hypermethylation in tumors (112, 113). Specifically, oxidative stress generates 7,8-dihydro-8-guanine, which recruits DNMT1 that interacts with MSH2-MSH6 protein and methylates DNA promoters (112–114). JAK2, which is also associated with CIMP, localizes to the nucleus, interacts with MSH2-MSH6 upon oxidative stress induction and helps drive oxidative stress-induced interaction of MSH2-MSH6 with DNMT1 and consequently, global methylation (115).

## 5 Clinical implications

### 5.1 Biomarker

CIMP has been found to independently associate with patient survival in several cancer types, including kidney renal clear cell carcinoma, hepatocellular carcinoma, leukemia, gastric cancer, breast cancer, and adrenocortical carcinoma (30, 32, 39, 42, 43, 53, 116–119). CIMP is associated with worse prognosis in colorectal cancer, breast cancer, renal cell carcinoma, and adrenocortical carcinoma (26, 30, 41–43, 118, 120–124). CIMP status is also associated with other cancer type-specific clinical characteristics. In colorectal cancer, CIMP-H status is associated with female gender, proximal tumor location, higher tumor grade, older age, poor differentiation, and MSI (23, 26, 117). Moreover, colorectal cancer diagnosed within 5 years after colonoscopy is more likely to have CIMP and MSI than cancer diagnosed after 5 years (125). In breast cancer, CIMP is associated with high grade and increased metastatic risk (53, 118). Further, methylation in serum is also associated with breast cancer and recurrence risk of rural sporadic breast cancer, showing potential of CIMP to be detectable in serum of breast cancer patients and allow distinction between tumor and normal samples with at least 90% specificity and sensitivity (35, 36, 126). Classification of glioma based on combination of CIMP status and copy number alteration status is associated with survival (63, 68, 69). In addition, CIMP is associated with better overall survival, as well as low-grade glioma and improved outcome (63–65, 68, 69, 127, 128). Studies have also found that upon recurrence, there is a shift from CIMP high to CIMP low (26, 41).

However, clinical features of CIMP in gastric cancer and leukemia remain ambiguous. Some studies have shown that CIMP is associated with better overall survival and progression-free survival while others have concluded that CIMP is associated with higher stage, lymph node metastasis, and worse survival (27–34, 49, 120, 121, 129). This discrepancy is likely due to heterogeneity between studies in both CIMP markers and patient samples. Majority of studies showing better prognosis identifies CIMP by a set of genes that included *MINT1*, *MINT2*, *MINT12*, *MINT25*, and *MINT31*. In acute myeloid leukemia, some studies have found A-CIMP patients are associated with longer overall survival than CIMP-negative patients while I-CIMP patients are not (59, 130). There is also evidence of increase in methylation at relapse (38). Contrarily, recent studies using genome-wide approaches have found CIMP patients are associated with better overall and disease-free survival in both T and B cell acute lymphoblastic leukemia, with shorter response to treatments in T cell acute lymphoblastic leukemia (42, 43, 131, 132). Clinical features of CIMP acute lymphoblastic leukemia are ambiguous, possibly also due to the lack of consensus on the definition of CIMP. Early studies with CIMP defined by selective CpG sites found CIMP patients are associated with worse disease-free and overall survival (37, 39). Similarly, inconclusive results from gastric cancer perhaps are due to differences in what genes are used to identify CIMP, which further warrants meta-analysis of global methylation data in the future to identify CIMP. Regardless, specific differentially methylated regions can also be used to distinguish different subtypes (50, 53, 133). In gastric cancer, CIMP, as defined within each study, is associated with EBV, methylation increases with tumor progression, and CIMP status in combination with *TP53* hotspot mutation status forms subgroups with distinct overall and progression free survival (28, 29, 31, 34, 47, 134).

In prostate cancer, the hypermethylator phenotype is associated with clinical features of poor prognosis. Multiple genes, such as *GSTP1*, *APC*, *MDR1*, *MGMT*, and *RASSF1A*, show higher methylation frequency in prostate cancer samples compared to BPH and non-neoplastic prostate samples (74, 78). Additionally, high methylation of *RARβ*, *RASSF1A*, *GSTP1*, *CDH13*, *APC*, *RUNX3*, *MDR1*, and *cyclin D2* is associated with high Gleason score and high PSA (74–76, 78). Methylation score, determined by statistical analysis comparing methylation status of various genes of benign prostatic hyperplasia and prostate cancer, is also found to be associated with high pT and other advanced pathological features, and can also distinguish organ-confined cancers from locally advanced cancer (74). Through MS PCR, Yegnasubramanian et al. also show that hypermethylation patterns of *GSTP1*, *APC*, *RASSF1A*, *PTGS2*, and *MDR1* is able to be used to distinguish primary prostate cancer from benign prostate tissues, hypermethylation of CpG island at *EDNRB* is correlated with tumor grade and stage of primary prostate cancers, and hypermethylation of CpG island of *PTGS2* is associated with increased risk of recurrence (135). Beyond these specific loci, analysis using Illumina HumanMethylation27 platform has identified 87 CpG sites with increased DNA methylation in 83/87 tumor samples, making them the most predictive diagnostic methylation biomarkers that can predict either tumor state or benign adjacent state of prostate cancer (101). Also, by integrating clinical follow-up data, it has been shown that there are prognostic DNA methylation alterations that correlate with biochemical recurrence of tumor (101). Furthermore, hypermethylation changes are highly maintained across anatomically distinct metastases within an individual, highlighting the potential of methylation status to be used as a longitudinal biomarker for clinically advanced prostate cancer (70). Methylation patterns have also been shown to be able to distinguish between castration resistant adenocarcinoma and neuroendocrine prostate cancer, and Berchuck et al. has been able to build a model that predicts the presence of NEPC using MeDIP-seq with 100% sensitivity and 90% specificity (5, 136). Furthermore, methylation changes have also been detectable in cell-free DNA (cfDNA), showing potential of methylation patterns to be used to develop liquid biomarkers. Liquid biomarkers, including circulating tumor cells, tumor cell fragments, nucleic acids, and proteins, are more readily accessible through any bodily fluids such as urine and blood, making them more easily obtainable than biopsies of prostate cancer metastases. As such, ability to detect methylation patterns in cfDNA and use it to distinguish specific clinical features holds significant clinical implications. To do so, cfDNA are isolated from plasma samples of patients with localized and metastatic prostate cancer, isolated, then profiled using bisulfite sequencing, MeDIPseq, or 5hmC sequencing (5hmC-seq). cfDNA global methylation patterns within each individual are temporally stable throughout the disease course, can distinguish metastatic from localized samples with 0.989 prediction accuracy, and can be used to build a model that can predict presence of NEPC and discriminate NEPC from castration resistant prostate adenocarcinoma (82, 87, 136). Moreover, using methylation sensitive restriction enzyme-qPCR analyses in liquid biopsies from mCRPC patients responsive and non-responsive to different therapies, Dillinger et al. has found higher methylation of specific loci in non-responsive patients before and after abiraterone treatment and identified 23 individual marker genes for which methylation was a negative prognostic factor for disease recurrence (137). In addition, Wu et al. have shown by sequencing plasma DNA from mCRPC patients receiving abiraterone or enzalutamide pre and post chemotherapy, there is hypomethylation of segments of AR binding sequences that are associated with AR copy number gain and more aggressive clinical course (138). And as previously mentioned, 5hmC patterns with prognostic value can also be detected in cfDNA of mCRPC patients (88). Overall, methylation alterations show potential, even as a liquid biopsy, to serve to work in conjunction with genetic alterations for clinical biomarker development.

Using bisulfite sequencing, MeDIP-seq, or hMeDIP-seq are useful tools for scientific research. It allows exploration of mechanisms and its potential as a clinical prognostic biomarker. However, to be used in a clinical setting, the cost for running these sequencing methods should be considered. Currently, there is effort to develop targeted panels of DNA methylation to help reduce cost. In addition, there are commercial efforts to use different methods beyond pulling down methylated or hydroxymethylated DNA using antibodies to analyze DNA methylation patterns (139).

### 5.2 Treatment options

As CIMP tumors are hypermethylated, several DNA methyltransferase inhibitors, such as decitabine and azacitidine, are being evaluated in pre-clinical and clinical settings (34, 128, 140–142). DNA methyltransferase inhibitors azacitidine and decitabine are FDA-approved and show potential as a new therapeutic anticancer treatment. Treatment with decitabine has shown to slow tumor growth, decrease cell proliferation, and induce tumor suppressors in breast cancer cell lines in *in vitro* and *in vivo* studies using mice induced with human breast cancer cell lines (141, 142). Similarly, decitabine administered with talazoparib decreases tumor growth and increases overall survival in ovarian and breast cancer models (140). Further, in gastric cancer, as EBV-induced hypermethylation targets and silences key tumor suppressor genes including *APC*, *RASSF1*, *BRCA1*, *THBS1*, and *CDKN2A*, DNA methyltransferase inhibitors may also serve as a new therapeutic treatment for patients with EBV-positive gastric cancer (34, 129). There are also some concerns with using demethylation through the use of DNA methyltransferase inhibitors as a new treatment method, as demethylation induces pro-metastatic genes and increases invasiveness of non-invasive breast cancer (141, 142). However, an *in vitro* study by Chik et al. shows that depletion of DNMT1 suppresses cell growth but does not induce invasiveness while depletion of DNMT3a does not change cell transformation and increases cell invasiveness, demonstrating that specific DNMT1 inhibitors, azacitidine and decitabine, may avoid adverse effects (142). Current clinical trials on DNA methyltransferase inhibitors include studies on side effects and best dose of decitabine with nivolumab in treating colorectal cancer, efficacy of treatment of azacitidine in recurrent *IDH1*-mutant gliomas and finding maximum tolerated dose of azacitidine with capecitabine and oxaliplatin in treating metastatic colorectal cancer. They measure maximum tolerated dose, overall response rate, adverse events, and progression free and overall survival after treatment with decitabine or azacitidine for a month to 1 year.

Azacitidine has been explored as a new therapeutic drug for prostate cancer treatment in combination with chemotherapy or anti-androgen therapy. Azacitidine shows antiproliferative effects in 22Rv1 and PC3 cell lines, and *in vivo*, 0.8 mg/kg intraperitoneal injection of azacitidine reduced tumor proliferation and induced apoptosis in PC3 and 22Rv1 xenografts (143). Additionally, azacitidine shows synergistic effects with docetaxel and cisplatin, sensitizing both PC3 and 22Rv1 xenografts to docetaxel and cisplatin treatments and causing tumor growth delay without complete regression (143). This combination treatment was superior to either treatment alone and tolerable in mice (143). Azacitidine has also been tried in a clinical setting, to determine if it can reverse docetaxel resistance in mCRPC patients with disease progression during or within 6 months after cessation of minimum 6 weeks of docetaxel-based therapy (144). In this phase I/II study, azaciditine and docetaxel were alternately escalated with administration of prednisone. They found there was >50% decline in PSA in 10 out of 19 patients, favorable progression free survival and overall survival, and the common treatment-related adverse event was neutropenia (144). Furthermore, there is a Phase II trial to study effect of azacitidine in modulating PSA in patients continuing treatment with luteinizing hormone-releasing hormone and antiandrogen. This study plans to detect biological activity of azacitidine as well, by measuring fetal hemoglobin and plasma DNA methylation (145).

## 6 Discussion

Methylation changes in prostate cancer, including hypermethylation of CpG islands, hypomethylation patterns, and 5hmC patterns are reported in cancer transformation and progression. The CIMP subtype in prostate cancer shows decreased expression of tumor suppressor genes and has been associated with distinct clinical features, including higher Gleason score, higher PSA, higher tumor grade, and overall poor outcome. Tumors of this hypermethylation subtype can potentially benefit from FDA-approved demethylating agents, azacitidine and decitabine. There are also distinct patterns of methylation that can help distinguish benign prostate tissue from malignant prostate tumors, as well as the NEPC subtype from castration resistant adenocarcinoma. In addition, it can potentially be used to distinguish mCRPC. Further, the 5hmC landscape of prostate cancer also shows potential to serve as a marker of epigenetic activation throughout disease progression that can also identify distinct oncogenic signaling pathways that define subgroups of advanced prostate cancer and disease states. Prognostic DNA methylation patterns can also be detected in cell-free DNA isolated from plasma of patients with prostate cancer. Since biopsies of prostate cancer metastases can be difficult to obtain in comparison to more readily accessible plasma, analysis of methylation patterns in cfDNA can add to current analyses of cfDNA in advanced cancers to serve as a better liquid biomarker. Studies into methylation patterns in prostate cancer have also been improved with novel methods, such as whole genome bisulfite sequencing and MeDIP sequencing. Inclusion of DNA methylation data into future multi-omic studies of prostate cancer patient samples of different stages and clinical subtypes will allow better understanding of the molecular heterogeneity of prostate cancer. Delineating the relationship between the driving mutations (e.g. *DNMT*, *IDH*, *TET*, and *BRAF* genes) and aberrant methylation patterns in PCa can underlie the complex mechanism and help predict specific methylation subtypes. Future studies integrating methylation sequencing data with sequencing investigating chromatin structure such as chromatin immunoprecipitation sequencing (ChIP-seq) and chromatin interaction analysis by paired-end tag sequencing (ChIA-PET), could reveal complex 3D epigenetic regulation. Overall, DNA methylation analysis not only could elucidate mechanisms that drive cancer progression but also demonstrate potential for clinical biomarker and novel treatment plan development for prostate cancer.

## Author contributions

HS, JH, and HL drafted the article or revised it critically for important intellectual content. All authors contributed to the article and approved the submitted version.
